# BET inhibition as a single or combined therapeutic approach in primary paediatric B-precursor acute lymphoblastic leukaemia

**DOI:** 10.1038/bcj.2013.24

**Published:** 2013-07-19

**Authors:** D Da Costa, A Agathanggelou, T Perry, V Weston, E Petermann, A Zlatanou, C Oldreive, W Wei, G Stewart, J Longman, E Smith, P Kearns, S Knapp, T Stankovic

**Affiliations:** 1 School of Cancer Sciences, University of Birmingham, Birmingham, UK; 2 Department of Clinical Medicine, Structural Genomics Consortium, University of Oxford, Oxford, UK; 3 Birmingham Children's Hospital, Birmingham, UK

**Keywords:** BET proteins, inhibitor, ALL

## Abstract

Paediatric B-precursor ALL is a highly curable disease, however, treatment resistance in some patients and the long-term toxic effects of current therapies pose the need for more targeted therapeutic approaches. We addressed the cytotoxic effect of JQ1, a highly selective inhibitor against the transcriptional regulators, bromodomain and extra-terminal (BET) family of proteins, in paediatric ALL. We showed a potent *in vitro* cytotoxic response of a panel of primary ALL to JQ1, independent of their prognostic features but dependent on high *MYC* expression and coupled with transcriptional downregulation of multiple pro-survival pathways. In agreement with earlier studies, JQ1 induced cell cycle arrest. Here we show that BET inhibition also reduced c-Myc protein stability and suppressed progression of DNA replication forks in ALL cells. Consistent with c-Myc depletion and downregulation of pro-survival pathways JQ1 sensitised primary ALL samples to the classic ALL therapeutic agent dexamethasone. Finally, we demonstrated that JQ1 reduces ALL growth in ALL xenograft models, both as a single agent and in combination with dexamethasone. We conclude that targeting BET proteins should be considered as a new therapeutic strategy for the treatment of paediatric ALL and particularly those cases that exhibit suboptimal responses to standard treatment.

## Introduction

Over the last few decades, a significant improvement in the treatment of children with paediatric B precursor acute lymphoblastic leukaemia (B-precursor ALL) has been achieved, mostly due to the rational use of intensive chemotherapy, and careful clinical and molecular stratification.^1, 2, 3^ Despite this, a proportion of patients still experience disease progression, suggesting a heterogeneous disease pathogenesis. In addition, a number of patients suffer from debilitating long-term post-chemotherapy effects,^4, 5^ and consequently, there is an obvious requirement for more targeted therapeutic approaches.

The mechanisms underlying chemoresistance and disease progression in paediatric ALL are still poorly understood. Published data suggest that in a proportion of patients, progressive disease is associated with the presence of *bcr/abl* fusion transcripts, *IKZF1* mutations and deletions, activating *JAK* mutations, or alterations involving cytokine receptor-like factor 2.^6, 7, 8, 9, 10, 11, 12^ In our previous studies, we have reported an association between apoptotic resistance to ionising radiation (IR)-induced DNA damage *in vitro* and clinical response *in vivo* measured by minimal residual disease (MRD).^13, 14^ Furthermore, we observed a connection between apoptotic resistance and transcriptional deregulation of multiple pro-survival pathways occurring in a leukaemia-specific manner. Finally, we noticed that pharmacological inhibition of individual pro-survival pathways led to heterogeneous responses *in vitro* and, therefore, does not provide a uniform strategy for counteracting apoptotic resistance.^13^

With this in mind, we focused on a therapeutic approach that has the potential to induce cellular death in a range of ALLs with different phenotypes. The bromodomain and extra-terminal (BET) family of proteins recently emerged as a new therapeutic target in haemopoietic malignancies. This family includes four proteins (BRD2, BRD3, BRD4 and BRDT) that recognise epigenetic chromatin modifications, such as polyacetylated lysine residues of histone tails, and form part of transcription complexes. BRD4 remains bound to transcriptional start sites of genes expressed during mitosis and affects the transcription of growth- and survival-promoting genes.^15^ Interestingly, a recent screen of an short hairpin RNA library targeting known epigenetic modifiers has identified BRD4 as the main factor that supports the maintenance of acute myeloid leukaemia (AML) stem cells.^16^

A pharmacological inhibitor, JQ1, with high target potency against BET proteins has recently been synthesised.^17^ This inhibitor has demonstrated striking anti-tumour activity *in vitro* and in pre-clinical murine models of c-Myc dependent malignancies such as aggressive nuclear protein in testis-midline carcinoma, AML and multiple myeloma.^17, 18, 19^ Furthermore, similar effects were observed in AML cells harbouring *MLL* fusion genes treated with another inhibitor against BET proteins, I-BET151.^20^ It has been proposed that the main mechanism of JQ1-induced cytotoxicity involves downregulation of c-Myc as well as Bcl-2; a principal anti-apoptotic protein.^16, 17, 18, 19^ A more recent study demonstrated JQ1-induced cytotoxicity in ALL cell lines via inhibition of the interleukin-7 (IL-7) receptor and JAK/STAT signalling pathways.^21^

Taken together, it seems plausible that BET inhibition induces multiple cellular effects and that the cytotoxic mechanism in different haemopoietic malignancies depends on their distinctive molecular phenotypes. Although a recent report suggests that ALL might be another haemopoietic malignancy where BET inhibition is therapeutically beneficial,^21^ a comprehensive study on primary ALLs with different features has not been performed. Therefore, the purpose of this study was to address sensitivity to BET inhibition in a wide range of primary paediatric ALL samples and expand current understanding of its cellular effects and the mechanistic aspects of BET inhibition, with a view of optimising the future therapeutic application of this promising therapeutic strategy.

We analysed the sensitivity of 26 paediatric primary B-precursor ALL samples with different clinical and molecular features to JQ1 *in vitro* and observed a cytotoxic effect irrespective of their phenotype. Similarly, we noted a complete suppression of ALL tumour growth in two different xenograft models in NOG mice. In primary ALL cells, global gene expression profiling revealed JQ1-induced downregulation of multiple pro-survival pathways and a therapeutic response dependent on the basal expression of cell cycle regulators c-Myc and its target p21. JQ1 induced cell cycle arrest and apoptosis, as well as increased replication fork stalling. Consistent with these findings, co-treatment of JQ1 with dexamethasone further suppressed leukaemic cells both *in vitro* and *in vivo* in xenografts created from both a primary ALL and a dexamethasone-resistant ALL cell line.

## Materials and methods

### Cell lines and patient samples

We analysed the B-precursor ALL cell lines NALM-6, NALM-17, REH, SUPB-15, TOM-1, SD1, the T-ALL Jurkat and bone marrow samples from 26 children with B-precursor ALL and two paediatric AML (Supplementary Table 1). Diagnosis of primary B-precursor ALL or AML was established by standard morphological and immunophenotypic evaluation. Written consent was obtained from all patients and control individuals.

### Cell viability assays

Viability was assessed using the CellTiter-Glo Luminescent Cell Viability Assay (Promega, Southampton, UK), according to the manufacturer's instructions.

### Protein expression analysis

Western blotting was carried out as previously described^14^ with primary antibodies: rabbit anti-PARP1 and mouse anti-procaspase-7 (Cell Signaling, Boston, MA, USA), mouse anti-c-Myc (Calbiochem, San Diego, CA, USA), rabbit anti-BRD4, anti-BIRC5 and mouse anti-Bcl-2 (Santa Cruz Biotechnology, Santa Cruz, CA, USA), mouse anti-p53 (DO1) (Roger Grand, University of Birmingham, UK), rabbit anti-phospho-p53 Ser15 (Bethyl Laboratories, Montgomery, TX, USA), rabbit anti-Aurora Kinase A and anti-p21 (Abcam, Cambridge, UK), goat anti-BIRC3 (RD Systems, Tustin, CA, USA), mouse anti-Mcl-1 (BD Pharmingen, San Diego, CA, USA) and mouse anti-beta-actin (Sigma-Aldrich, St Louis, MO, USA).

Immunohistochemistry of engrafted organs embedded in paraffin blocks was performed according to the standard protocols with a specific antibody against c-myc.

### Microarray analysis

RNA preparation and hybridisation to Affymetrix Human Gene 1.0ST Arrays was performed in eight primary ALL samples both before and after 6 h treatment with 1 μℳ JQ1 according to the manufacturer's protocol (Affymetrix, Santa Clara, CA, USA).

Gene expression analysis of the array data was carried out using the Affymetrix Expression Console with default settings of ‘Default: RMA-Sketch'. Differentially expressed genes with a *P*-value <0.001 and fold change >2 were identified using limma analysis.^22^ Gene set enrichment analysis^23^ was carried out with the javaGSEA Desktop Application (http://www.broadinstitute.org/gsea/). To determine genes predictive of response to JQ1, ranking according to EC50 value and Spearman's rank correlation coefficient analysis was performed in untreated, basal, ALL samples. This identified a set of genes that was subsequently subjected to cytoscape analysis (http://www.cytoscape.org/) to identify cellular pathways influencing the level of JQ1-induced cellular death.

### Cell cycle and DNA fibre analysis

Cell cycle was analysed by fluorescence-activated cell sorting analysis of BrdU and propidium iodide-labelled cells.^24^ Briefly, 3 × 10^6^ NALM-6 cells were pulse-labelled with 10 μℳ BrdU, fixed in ethanol, incubated in 15 mℳ pepsin and 2 N HCl before incubation with a mouse anti-BrdU antibody (Dako, Cambridge, UK) and appropriate secondary antibody (Vector Laboratories, Peterborough, UK). For total DNA staining, cells were stained with propidium iodide.

Replication tract abnormalities were detected by the previously described DNA fibre technique^25^ to visualise the effect of JQ1 on DNA replication. Ongoing replication forks in proliferating cells were pulse-labelled with two different thymidine analogues, 5-chloro-2′-deoxyuridine and 5-iodo-2′-deoxyuridine, which are incorporated into newly synthesised DNA. Subsequent differential immunostaining with specific antibodies enabled the visualisation of different replication intermediates such as replication origins, ongoing replication forks and terminated or stalled forks, as well as measurement of the speed of ongoing replication forks.

For dual labelling of replication tracts, exponential cell cultures were pulse-labelled with 25 μℳ CIdU (Sigma-Aldrich) followed by 250 μℳ IdU (Sigma-Aldrich), for 20 min each. For immunodetection of CIdU- and IdU-labelled tracts, acid-treated fibre spreads were incubated with a mix of rat anti-BrdU monoclonal antibody, clone BU1/75 (ICR1) (Serotec, Oxford, UK) and mouse anti-BrdU monoclonal antibody (Becton Dickinson, Oxford, UK). Slides were fixed in 4% paraformaldehyde (Sigma) and incubated with a mix of AlexaFluor 555-conjugated goat anti-rat and AlexaFluor 488-conjugated goat anti-mouse immunoglobulin G (IgG) (Molecular Probes, Eugene, OR, USA). Fibres were examined using a Nikon, (Melville, NY, USA) Eclipse E600 microscope with a × 60 lens and images acquired using the Volocity package (Perkin Elmer, Hopkinton, MA, USA). For replication fork speeds, the lengths of red- (CIdU-AlexaFluor 555) and green- (IdU-AlexaFluor 488) labelled patches were measured using the ImageJ software (http://rsb.info.nih.gov/ij/). Length values were converted into kb using the conversion factor 1 μm=2.59 kb. Replication structures as shown in Figure 6a were counted using the Cell Counter plugin for ImageJ.

### ALL xenograft models

Animals were treated in accordance with the UK Home Office guidelines, Schedule 1. Subcutaneous tumour cell line xenograft models were generated following injection of 1 × 10^6^ cells subcutaneously into the left flank of NOG mice. Primary ALL xenografts were generated by intravenous injection of 1 × 10^6^ tumour peripheral blood mononuclear cells into NOG mice. Intraperitoneal treatments commenced upon evidence of ⩾1% human CD45+ leukaemic cells in peripheral blood.

## Results

### JQ1 has cytotoxic effect in ALL cell lines and primary ALL both *in vitro* and *in vivo*

**Figure 1:**
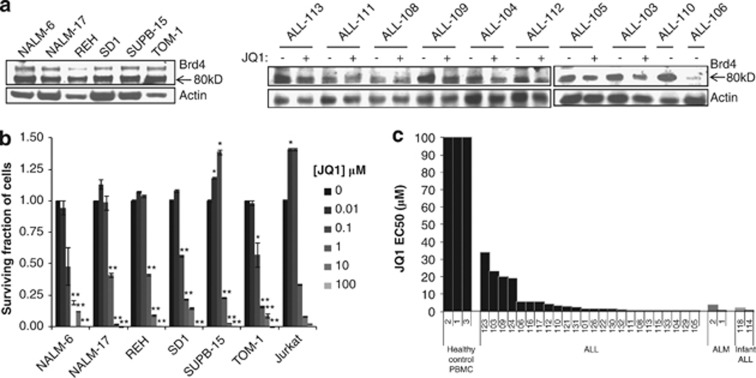
ALL cell lines and primary tumours exhibit sensitivity to JQ1 *in vitro*. (**a**) Western blots showing expression of the 80-kDa BRD4 protein in B-precursor ALL cell lines, NALM-6, NALM-17, REH, SD1, SUPB-15 and TOM-1 (left). Ten representative primary B-precursor ALL samples show Brd4 expression levels that remained mostly unchanged following 6 h exposure to 1 μℳ JQ1 (right). (**b**) Cell lines NALM-6, NALM-17, REH, SD1, SUPB-15, TOM-1 and Jurkat were seeded into opaque 96-well plates (5 × 10^4^ cells per well) and cultured in the presence or absence of increasing concentrations of JQ1 (0.001–100 μℳ). Cell lines incubated with ⩾1 μℳ JQ1 for 72 h show a dramatic loss of viability. Low drug doses can result in a phenomenon called compensatory proliferation as observed here for SUPB-15 and Jurkat.^42, 43^ Data are presented as mean±s.e.m., and statistical significance was determined by paired, two-tailed Student's *t*-test (*P⩽0.05, **P⩽0.005). (**c**) Compared with peripheral blood mononuclear cell obtained from three healthy individuals, representative primary ALL and AML samples show a differential loss of viability upon 48 h incubation with JQ1. Data are presented as JQ1 concentration required to affect 50% of cells (EC50) determined using Calcusyn Version 2.1 for Windows software (Biosoft, Paolo Alto, CA, USA).

We first observed that the level of expression of BET protein BRD4 was uniform in representative ALL cell lines and primary ALL samples. Consistent with an effect on BRD4 activity rather than transcription, JQ1 had no impact on BRD4 expression in primary ALL cells (Figure 1a). We subsequently determined cell viability following 72 h exposure to increasing doses of JQ1 in a panel of ALL cell lines including Ph^+^ ALL (SUPB-15, SD1, TOM-1), Ph^−^ ALL (REH) that exhibit a defective apoptotic response to IR *in vitro* and Ph^−^ (NALM-6, NALM-17) with a normal apoptotic response to IR, as well as T-ALL (Jurkat). An impressive decrease in cell viability was observed in all cell lines tested, with EC50 values <850 nℳ (Figure 1b).

In comparison, 26 primary B-precursor ALL samples showed a range of cytotoxic responses with EC50 values <10 μℳ for the majority of tumours (20/26) (Figure 1c). These sensitive cases exhibited cytogenetic abnormalities commonly observed in paediatric or infant ALL, such as hyperdiploidy, t(12;21), t(1;19) and *MLL* rearrangements (Supplementary Table 1), and represented a mixture of leukaemias with high or low MRD risk after induction treatment. No association was observed between any particular cytogenetic abnormality or MRD risk and JQ1 response. Notably, four primary leukaemias, one with high-risk MRD (ALL-123) and three with low risk MRD (ALLs 124, 109 and 102), were less responsive to JQ1 (EC50 ⩾19 μℳ). Consistent with previous reports,^16, 19, 20^ primary paediatric AML samples were sensitive to JQ1 (EC50 1.08±0.75), whereas in control peripheral blood mononuclear cells, cytotoxicity could not be observed for the same dose range (EC50 >800 μℳ).

**Figure 2:**
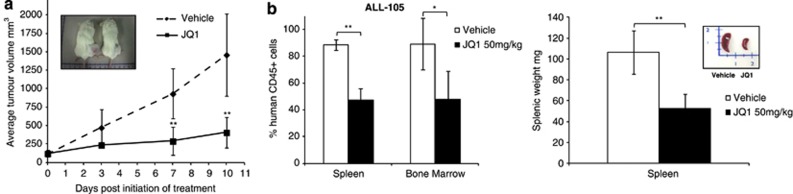
ALL tumour cells exhibit sensitivity to JQ1 *in vivo*. (**a**) Subcutaneous NALM-6 tumours show significant impairment of growth upon treatment with 50 mg/kg JQ1 (*n*=8) for 5 days a week over a total of 2 weeks compared with tumours treated with vehicle (*n*=8). This difference in tumour size is shown in the photograph of two representative animals, treated with vehicle (10% (w/v) 2-hydroxy-propyl-β-cyclodextrin, Sigma-Aldrich) or JQ1 respectively (top left-hand inset). Intraperitoneal treatments commenced upon evidence of visible tumours, and tumours were measured thrice weekly by calliper. (**b**) In a xenograft of a primary sample, ALL-105 treatment with 50 mg/kg JQ1 (*n*=7) for 5 days a week over 4 weeks leads to a visible reduction in engrafted spleen size/weight (inset and right-hand panel), accompanied by a significant reduction of splenic engraftment (middle panel) and therefore reduced tumour load compared with vehicle treatment as assessed by fluorescence-activated cell sorting analysis (*n*=7). The remainder of cells are of murine origin. Data are presented as mean±s.d., and statistical significance was determined by unpaired, two-tailed Student's *t*-test (*P⩽0.05, **P⩽0.005).

We proceeded to test the impact of JQ1 on ALL proliferation *in vivo*. Following formation of subcutaneous tumours of the ALL cell line NALM-6, animals received either JQ1 or vehicle alone. We observed a dramatic reduction of tumour growth in JQ1-treated animals, compared with those given vehicle treatment (Figure 2a). This was accompanied by prolonged event-free survival as JQ1-treated tumours never attained 1500 mm^3^ tumour size over the experimental time course (Supplementary Figure 1A).

Similarly, JQ1 treatment induced both a dramatic reduction of tumour load in a primary ALL xenograft model created from a high-risk primary B-precursor ALL (ALL-105) (Figure 2b) and extended event-free survival (⩾25% hCD45+ cells in peripheral blood) by at least 24 days (Supplementary Figure 1B). The engrafted leukaemia revealed the presence of early CD34+CD19+CD10−, intermediate CD34+CD19+CD10+ and late CD34−CD19+CD10+ ALL progenitors that had the capacity to proliferate *in vivo*. We also observed a small percentage of CD34−CD19+CD10− cells in engrafted spleens, whose malignant origin was uncertain. We noted JQ1-induced reduction of all the three ALL progenitors, with the greatest effect being exerted on the CD34+CD19+CD10+ subset (Supplementary Figure 1C).

We conclude that JQ1 exhibits a cytotoxic effect on ALL cells both *in vitro* and *in vivo* and that this effect is independent of the ALL phenotype.

### JQ1 targets primary ALL cells with high *MYC* levels and downregulates multiple pro-survival pathways

**Figure 3:**
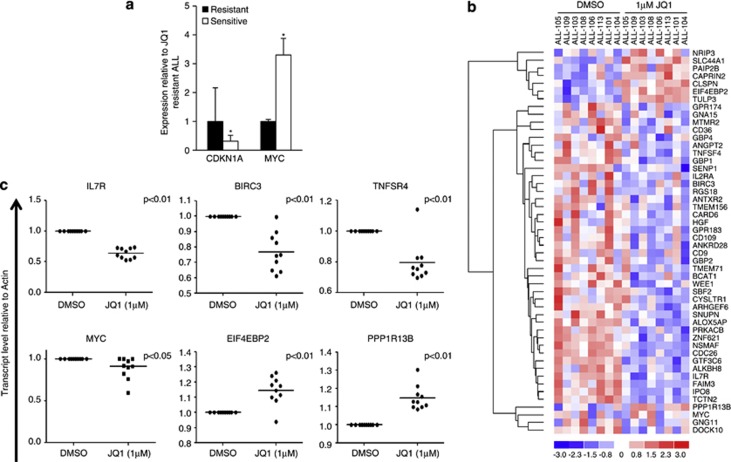
JQ1 targets primary ALL cells with high *MYC* expression and downregulates multiple pro-survival pathways. (**a**) SYBR Green quantitative reverse transcriptase PCR (Q-PCR) was performed using 7500 Fast Real Time PCR system (Applied Biosystems, Warrington, UK), and comparative Ct method^44^ was applied to normalise and quantify expression levels. Primer sequences are available on request. Q-PCR shows differential expression of *CDKN1A* and *MYC* in JQ1-resistant (EC50 >10 μℳ) versus JQ1-sensitive (EC50 <10 μℳ) primary ALLs (total *n*=12). Data are expressed relative to JQ1-resistant samples, and the differences in mRNA levels were analysed by a one-tailed *t*-test (*P⩽0.05). (**b**) Heat-map, generated using dChip (http://www.dchip.org/) with the default settings, shows a selected subset of genes downregulated or upregulated in eight primary ALLs by 6 h treatment with 1 μℳ JQ1. Downregulated genes belong to multiple pro-survival pathways and include c-Myc targets, the JAK/STAT pathway, and also members of the interleukin gene family. Each column represents a different patient sample and each row represents a single gene. Colour changes within a row indicate expression levels relative to the average of the same population. Red indicates upregulation and blue indicates downregulation. (**c**) Downregulation (*IL7R*, *BIRC3*, *TNFSR4*, *MYC*) or upregulation (*PPP1R13B*, *EIF4EBP2*) of representative genes induced by 6 h treatment with 1 μℳ JQ1 is confirmed by Q-PCR in a selection of primary ALL samples (*n*=10), some included and some not in the microarray analysis. JQ1 causes a modest reduction in *MYC* mRNA levels. The differences in mRNA levels were analysed by a two-tailed *t*-test.

Given that clinical and cytogenetic profiles of primary ALL samples did not influence the response to JQ1, we addressed whether their global gene expression profiles could provide additional information. Spearman's rank correlation was implemented on global transcription profiles of eight untreated, representative ALL cases with a range of cytotoxic responses to JQ1, and identified a set of 398 genes whose basal expression correlated with JQ1 EC50 values (Supplementary Table 2). Subsequent cytoscape analysis to identify common cellular functions highlighted two major pathways predictive of cytotoxic response to JQ1; one associated with *ATM* and related double-strand break repair genes, and the other linked with the cell cycle inhibitor *CDKN1A* (Supplementary Figure 2). We focused on the cell cycle inhibitor *CDKN1A* mRNA, and in a quantitative PCR validation experiment, we demonstrated that *CDKN1A* expression was significantly lower in ALL samples with low JQ1 EC50 values, whereas it's upstream negative regulator *MYC* was significantly higher in the same category of leukaemias (Figure 3a). We conclude that high *MYC* and low *CDKN1A* levels, likely to reflect an imprint of prior high proliferation in patients, render primary ALLs sensitive to BET inhibition.

To fully understand the JQ1-induced cytotoxic activity in primary paediatric ALL and identify the additional pathways that might be affected by BET inhibition, we next analysed JQ1-induced changes in global gene expression in the same eight primary ALL samples. Comparison of profiles before and after 6 h exposure to 1 μℳ JQ1 identified a total of 96 differentially downregulated genes in JQ1-treated cells. Consistent with a recently published report on ALL cell lines,^21^ we also observed downregulation of the IL-7 receptor gene (*IL7R*) that mediates pro-survival signalling via the JAK/STAT pathway (Figures 3b and c). Additional downregulated genes included those involved in the inhibition of apoptosis, such as the NFkB target gene *BIRC3*, the *FAIM3* gene encoding the anti-apoptotic factor Toso, *SENP1-*, *ALKBH8-* and *CARD6-*encoding proteins associated with the promotion of cancer cell survival, and also the *MYC* gene, a key regulator of cell proliferation, which exhibited higher basal levels in the more JQ1-responsive leukaemias. Subsequent assessment of downregulated genes by gene set enrichment analysis revealed multiple pro-survival pathways, including members of the JAK-STAT, NFkB, c-Myc and cytokine (IL-2, IL-7, IL-10, IL-17), and G-protein-coupled receptor pathways (Supplementary Table 3). A total of 127 genes were upregulated upon JQ1 treatment and included those with a role in translational repression (*EIF4EBP2*, *PAIP2B*), induction of apoptosis (*PPP1R13B*) and tumour suppression (glucocorticosteroid (GC)-responsive gene *FKBP51)* (Figure 3b).

We performed validation of genes relevant for ALL survival and proliferation, the pathways identified as being deregulated by JQ1, in a cohort that included ALL samples previously interrogated by microarray analysis, but also in those that were not analysed but were representative of the heterogenous nature of this malignancy. We confirmed a moderate decrease in *MYC* expression and a significant reduction (*IL7R*, *BIRC3*, *TNFSR4*) or elevation (*PPP1R13B* and *EIF4EBP2*) in the expression of these selected genes at the cytotoxic dose of JQ1 (Figure 3c).

**Figure 4:**
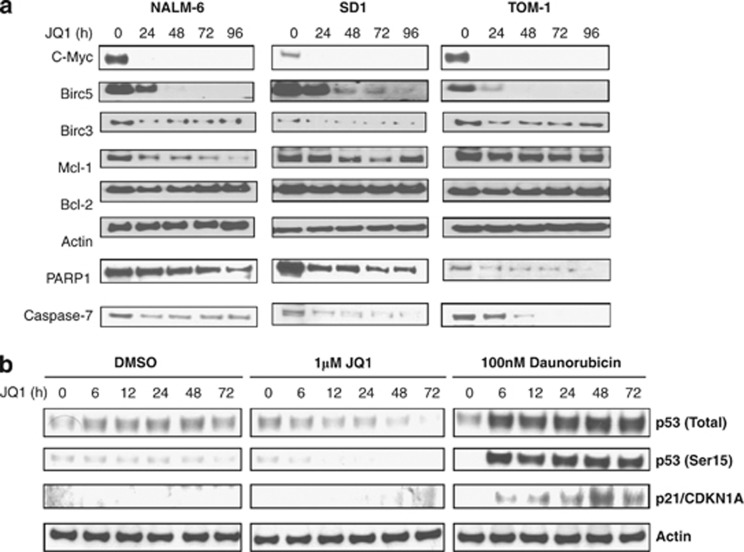
JQ1 reduces pro-survival protein expression and induces apoptosis. (**a**) Western blots showing, in addition to c-Myc, uniform downregulation of the pro-survival protein Birc5, variable downregulation of Mcl-1 and Birc3 and an unchanging expression of Bcl-2 in the ALL cell lines NALM-6, SD1 and TOM-1. Cleavage of the proteins PARP1 and caspase-7 over a 96-h incubation period with 1 μℳ JQ1 is suggestive of induction of apoptosis. Actin shows equal loading. (**b**) Western blots show no evidence of either p53 upregulation, p53 phosphorylation or significant p21 upregulation in NALM-6 cells incubated with 1 μℳ JQ1 over 72 h. In comparison, treatment with the known apoptosis-inducing, DNA-damaging agent, daunorubicin, induces expression of both total and phosphorylated p53, as well as expression of the p53 target p21.

Interestingly, when we addressed JQ1 effects at the protein level in ALL cell lines with a diversity of genetic backgrounds, we observed the most profound effect on both the cell cycle regulator c-Myc and the pro-survival protein BIRC5/survivin, which were completely abrogated in all cell lines tested by 48 h (Figure 4a). The effect on BIRC3 and Mcl-1 was variable between cell lines, whereas Bcl-2 levels remained unchanged.

Consistent with the ability of JQ1 to downregulate multiple pro-survival proteins, JQ1-treated cells exhibited evidence of p53-independent apoptosis. By 72 h of treatment, modest apoptosis could be detected by cleavage of PARP1, procaspase -7, -3 and by annexin V staining (Figure 4a, Supplementary Figure 3). In contrast to treatment with the DNA-damaging agent daunorubicin, JQ1-treated NALM-6 cells showed no evidence of p53 upregulation, p53 phosphorylation or p21 upregulation by western blot (Figure 4b).

### JQ1 decreases c-Myc stability

**Figure 5:**
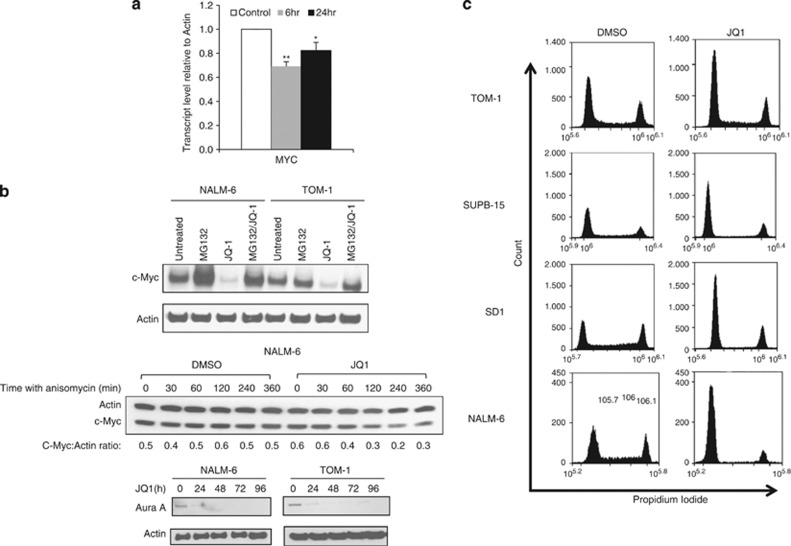
JQ1 downregulates *MYC* at both the transcriptional and the post-transcriptional level and induces cell cycle arrest. (**a**) Quantitative PCR showing a transient decrease of *MYC* mRNA in the cell line, NALM-6, following 6 h after treatment with 1 μℳ JQ1. Statistical significance was determined by a paired, two-tailed Student's *t*-test (*P⩽0.05, **P⩽0.005). (**b**) Western blots showing that proteasome inhibition with 10 μℳ proteasome inhibitor MG132 (Merck, Whitehouse, Station, NJ, USA) prevents downregulation of c-Myc after 24 h of incubation with 1 μℳ JQ1 in the ALL cell lines NALM-6 and TOM-1 (top). Inhibition of translation by 100 μℳ anisomycin (Sigma) over a 6-h time course in NALM-6 cells leads to accelerated decrease in c-Myc expression in the presence of JQ1 (c-Myc half-life is 2 h in JQ1-treated cells and >6 h in untreated cells) (middle). Aurora A kinase expression is depleted in NALM-6 and TOM-1 cell lines after 24 h of JQ1 treatment (bottom). (**c**) All four ALL cell lines (TOM-1, SUPB-15, SD1 and NALM-6) show an induction of G1 arrest following 24 h exposure to 1 μℳ JQ1 upon staining with 25 μg/ml propidium iodide (Sigma).

In agreement with published data, we found that although *MYC* mRNA levels were only moderately affected (Figures 3c and 5a), c-Myc protein was completely depleted by 24 h of JQ1 treatment in ALL cell lines (Figure 4a). Thus, we addressed the possibility that BET proteins might be involved in regulation of c-Myc stability. Consistent with this notion, BRD4 has been previously implicated in the regulation of the stability of papillomavirus-encoded E2 protein.^26^ We subsequently exposed NALM-6 and TOM-1 cells to 1 μℳ JQ1 for 24 h in the presence or absence of the proteasome inhibitor MG132 (Figure 5b) and observed that MG132 completely abolished JQ1-induced loss of c-Myc expression. Furthermore, inhibition of protein translation by anisomycin in NALM-6 cells led to an accelerated decrease in c-Myc expression in the presence of JQ1 (Figure 5b).

As c-Myc stability is regulated by Aurora A kinase,^27^ we addressed the possibility that JQ1-induced Aurora A downregulation might be associated with the loss of c-Myc expression. A concordant loss of c-Myc and Aurora A expression was detected in ALL cell lines by 24 h of treatment with JQ1 (Figure 5b). This suggests that JQ1 can indirectly modify c-Myc stability and stresses a potential role for BET proteins in the regulation of cellular proteins, not only at the transcriptional but also at the post-transcriptional level. As expected, JQ1-induced c-Myc downregulation was associated with G1 arrest in all ALL cell lines (Figure 5c).

### JQ1 inhibits DNA replication

As BET inhibition was able to reduce but not completely abolish cell cycle progression, we next investigated the effect of JQ1 on cells in S phase. Both c-Myc and BRD4 have been reported to have a role in DNA replication^28, 29^ and we reasoned that BET inhibition might compromise this highly regulated process, either directly or via its effect on c-Myc expression.

**Figure 6:**
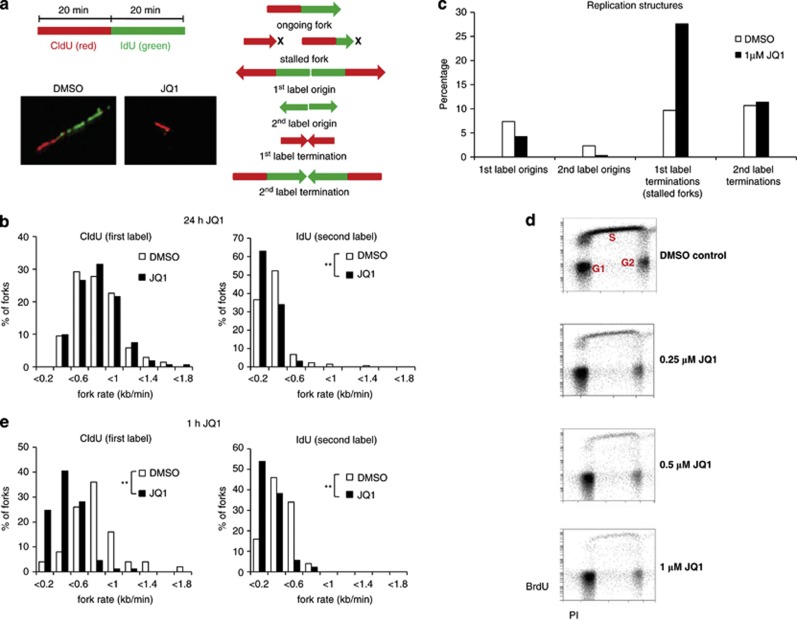
JQ1 inhibits DNA replication in ALL cells. (**a**) A schematic of DNA fibre labelling and representative images of replication forks in dimethyl sulphoxide- or JQ1-treated cells are given on the left, and a schematic representation of replication structures is given on the right. (**b**) The reduced lengths of IdU-labelled tracks reveal increased replication fork stalling in NALM-6 cells in the presence of 1 μℳ JQ1 over 24 h. (**c**) NALM-6 cells treated with 1 μℳ JQ1 over 24 h show an increased percentage of stalled replication forks. A representative assay is shown here. (**d**) BrdU incorporation analysed by fluorescence-activated cell sorting shows a normal S-phase cell distribution with a concordant reduction in the number of cycling cells in NALM-6 cells treated with increasing concentrations of JQ1. (**e**) Treatment of NALM-6 cells with JQ1 over 1 h also reduces progression of replication forks. Statistical significance was determined by a two-tailed Mann–Whitney test (**P⩽0.005).

We showed that 24 h exposure of NALM-6 cells to 1 μℳ JQ1 led to increased replication fork stalling as indicated by shortening specifically of the second label in ongoing forks (Figures 6a and b) and a substantial increase in the percentage of stalled replication fork structures (Figure 6c). To exclude the possibility that the observed effects on replication were caused by an accumulation of cells in a specific stage of the S phase, we measured BrdU incorporation in the same cell line (Figure 6d). We noted that JQ1-treated cultures exhibited a low proportion of S-phase cells, but those cells that had entered the S phase underwent DNA replication without accumulation in any particular S-phase stage. Furthermore, a short 1 h exposure of NALM-6 cells to 1 μℳ JQ1, insufficient to cause c-Myc downregulation, also led to a marked reduction in fork progression rates (Figure 6e). We conclude that reduced replication fork progression rates and increased replication fork stalling are caused by a direct effect of JQ1 on replication, rather than perturbation of the S phase itself, or by an indirect effect of c-Myc depletion.

### JQ1 sensitises ALL cells to dexamethasone, both *in vitro* and *in vivo*

The observed cellular consequences of JQ1 activity, namely the induction of G1 arrest, downregulation of pro-survival signalling and modulation of DNA replication provided a rationale for combined treatment.

**Figure 7:**
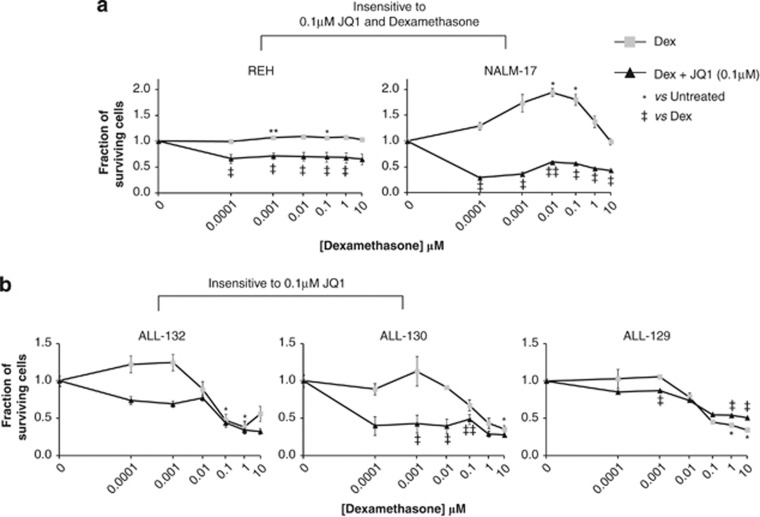
JQ1 sensitises ALL cells to dexamethasone *in vitro*. ALL cell lines and representative primary ALLs were incubated in triplicate with dexamethasone (0.0001–10 μℳ) in the presence or absence of 0.1 μℳ JQ1 for 72 h for cell lines and 48 h for primary ALLs. Where resistance to either one or both drugs was encountered (for example, REH and NALM-17), normalisation to either untreated controls for monotherapy or 0.1 μℳ JQ1-treated controls^45^ for dual therapy renders sensitisation to be visualised. This results in 0.1 μℳ JQ1 treatment alone being normalised to 1; thus any difference between dexamethasone alone vs co-treatment is evidence of some degree of sensitisation. Dexamethasone treatment appears to result in the phenomenon of compensatory proliferation^42, 43^ in NALM-17 cells. Data are presented as mean±s.e.m., and statistical significance was determined by paired, two-tailed Student's *t*-test (*^,‡^*P*⩽0.05, **^,‡‡^*P*⩽0.005). (**a**) Sensitisation to dexamethasone is evident in the dexamethasone-insensitive cell lines REH and NALM-17. (**b**) Primary tumours (ALL-130 and ALL-132) that are minimally responsive to the dose of JQ1 (0.1 μℳ) employed in these studies also show sensitisation to dexamethasone.

GCs are a principal component of current therapeutic protocols for paediatric ALL, and sensitivity to GCs is still the most important prognostic parameter in this type of leukaemia.^30^ It has been recently shown that dexamethasone induces cellular death by autophagy;^31^ however, the mechanisms underlying response to corticosteroids are complex and not fully understood.^30, 32, 33, 34^ Given that JQ1 downregulates multiple pro-survival genes that differ from GS targets^35^ (Supplementary Figure 4), we hypothesised that low doses of JQ1 would sensitise ALL cells to killing by dexamethasone. Indeed, significant sensitisation was observed *in vitro*, in two dexamethasone-resistant cell lines, NALM-17 and REH (Figure 7a). However, the synergistic relationship could be calculated for the two cell lines (NALM-6 and TOM-1) that were sensitive to both reagents. This revealed that JQ1 and dexamethasone also acted synergistically at a minimal level even in cell lines that responded well to both drugs individually, thus possibly allowing them to be used at doses that could reduce side effects of these individual therapies (Supplementary Table 4). Similarly, two primary ALLs (ALL-130 and ALL-132) could be sensitised to dexamethasone *in vitro* by addition of JQ1, whereas the effect in the highly JQ1-sensitive primary tumour (ALL-129) was less evident (Figure 7b, Supplementary Table 5).

**Figure 8:**
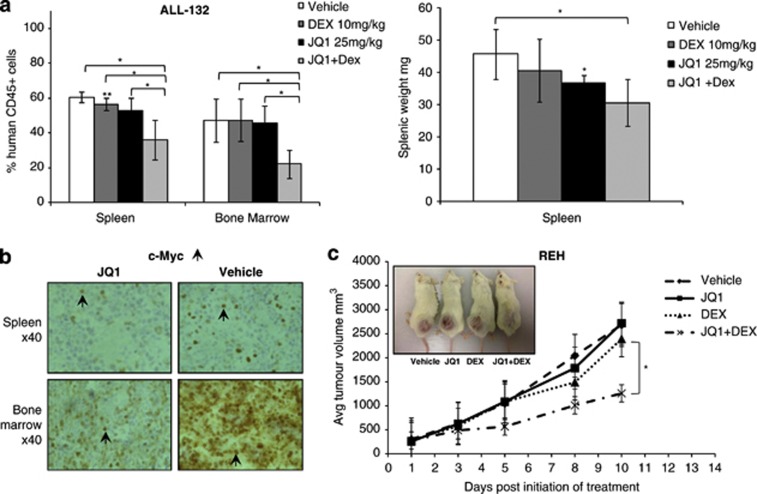
JQ1 in combination with dexamethasone reduces proliferation of ALL tumour cells *in vivo*. (**a**) In the primary xenograft model with ALL-132, intraperitoneal co-treatment (*n*=5) with JQ1 (25 mg/kg five times per week) and dexamethasone (10 mg/kg three times per week) for 3 weeks led to a significant reduction of engrafted cells both in infiltrated murine spleens and bone marrow compared with either dexamethasone or JQ1 treatment alone (*n*=4) (left-hand panel). The concomitant decrease in splenic size upon co-treatment indicates infiltration with fewer leukaemic cells and therefore reduced tumour load (right-hand panel). At this lower dose (25 vs 50 mg/kg), treatment with JQ1 alone (*n*=5) had no significant effect on tumour load compared with vehicle treatment (*n*=4). (**b**) Immunohistochemistry images reveal that JQ1 treatment of primary ALL xenografts resulted in downregulation of c-Myc in engrafted spleens and bone marrow. c-Myc-positive cells are stained brown as indicated by the arrows. (**c**) Subcutaneous REH tumours show significant reduction in growth when co-treated (*n*=4) with 25 mg/kg JQ1 (5 days a week) plus 10 mg/kg dexamethasone (thrice weekly) for 2 weeks compared with tumours treated with either vehicle (*n*=3) or dexamethasone alone (*n*=4). No significant effect on tumour growth was evident at this lower dose of JQ1 (*n*=3). Data are presented as mean±s.d., and comparisons were made by an unpaired, two-tailed *t*-test (*P⩽0.05, **P⩽0.005).

Finally, to address whether this *in vitro* sensitisation could be recapitulated *in vivo,* we treated a xenograft model of primary ALL-132 with a reduced dose of JQ1, with or without dexamethasone. Although this lower dose of JQ1 was insufficient to suppress leukaemic proliferation (Figure 8a), it led to downregulation of c-Myc (Figure 8b). In contrast, we observed a significant reduction in tumour load in animals co-treated with JQ1 and dexamethasone. Equally, in a xenograft model of the dexamethasone-resistant ALL cell line REH, we observed significantly reduced subcutaneous tumour growth under combined JQ1/dexamethasone treatment (Figure 8c). In addition, co-treatment conferred a survival advantage in both models (Supplementary Figure 5).

## Discussion

In this study, we presented a novel approach to the treatment of ALL that targets the histone acetyl-lysine-binding bromodomains of BET proteins, which regulate epigenetic memory and transcriptional activity. We showed that inhibition of BET proteins led to downregulation of multiple pro-survival genes, as well as cell cycle arrest and replication fork stalling. This caused cytotoxic effects in ALL cell lines as well as primary ALL cells irrespective of their phenotype, both *in vitro* and *in vivo* ALL xenograft models.

It has been reported that inhibition of the c-Myc-induced transcriptional program represents one of the principal mechanisms of cellular killing caused by BET inhibition.^17, 18, 19, 20^ Consequently, the BET inhibitor JQ1 suppresses c-Myc-driven malignancies, such as nuclear protein in testis-midline carcinoma, AML and multiple myeloma, by displacement of BRD4 from the *MYC* promoter/enhancer.^17, 18^ In this study, we have shown that BET inhibition downregulates not only *MYC* transcription but also reduces stability of c-Myc protein.

We addressed transcriptional markers of sensitivity to BET inhibition in primary ALL samples and observed that the cytotoxic response correlated with the expression of *MYC* and its target *CDKN1A* and that cases with the highest level of *MYC* expression exhibited low EC50 values. This is not surprising given the fundamental role of c-Myc downregulation in JQ1-induced cytotoxicity and suggests that highly proliferative and more aggressive ALL cases are more likely to be amenable to this new therapeutic strategy. Furthermore, as induction of apoptosis by JQ1 was not associated with p53 activation, there is a possibility that a wide range of apoptotic-resistant leukaemias, including those with p53 dysfunction, could be sensitive to BET inhibition. Most importantly, proliferation of ALL progenitors at different stages of differentiation were affected in JQ1-treated ALL xenografts.

Of note, we observed a differential sensitivity of ALL cells compared with non-leukaemia peripheral blood mononuclear cells. This was in agreement with a previous study, where short hairpin RNA suppression of BRD4 failed to influence the growth of non-transformed G1E erythroblast cells, despite a clear cytotoxic effect on AML cells.^16^

The question of whether the JQ1-induced cytotoxic effect can be entirely attributed to c-Myc downregulation remains intriguing. Several lines of evidence suggest that this might not be the case. For example, it has been previously observed that ectopic *MYC* expression was unable to prevent JQ1-induced cell death^16^ and that resistance to JQ1 can occur despite c-Myc downregulation.^19^ Our results support the notion that BET inhibition exerts an anti-tumour effect by a variety of mechanisms and that the cytotoxic effect of BET inhibition expands beyond c-Myc downregulation. First, we showed that in primary ALL cells, JQ1 led to substantial downregulation of different pro-survival pathways, including JAK/STAT and interleukin signalling components, that seem to be unaffected by BET inhibition in other malignancies studied so far. Second, our DNA fibre analysis suggests that JQ1-treated ALL cells in S phase exhibit not only reduced replication fork progression rates but also an increased frequency of stalled replication forks. This is consistent with a direct role for JQ1 in the induction of replicative stress. Although BrdU incorporation showed that JQ1-treated cells do not accumulate in S phase, this does not exclude the possibility that JQ1-treated cells might progress through S phase more slowly. At this stage, it is difficult to envisage the exact mechanism behind this defective replication; however, it is conceivable that upon BET inhibition, initiated but incompleted transcription complexes pose a physical obstacle for the replication process.^24^

BET inhibition sensitised both primary ALL and ALL cell lines to dexamethasone. GC sensitivity remains an important predictor of clinical outcome, and many European paediatric ALL trials include pre-treatment with GCs as an important stratifying step.^36, 37, 38, 39^ The survival advantage associated with dexamethasone over prednisolone in the treatment of ALL has been demonstrated in randomised controlled trials, although concerns remain regarding the relative toxicity.^40^ Thus, mechanisms to enhance the therapeutic efficacy of glucocorticoids would be of clinical interest.^41^ We observed JQ1-induced sensitisation to dexamethasone, both *in vitro* and *in vivo*, in two ALL xenograft models. The possibility that JQ1 and GCs target different pro-survival pathways in ALL cells could account for the observed sensitisation and would support this combination as a useful therapeutic approach for a wide range of ALL cases.

In conclusion, our results suggest that BET inhibition is an important novel target for paediatric ALL, irrespective of conventional prognostic features. We demonstrate that BET inhibition causes inhibition of the cell cycle and replication, repression of transcription and induction of apoptosis. Importantly, this effect could be enhanced by combining JQ1 with dexamethasone. Our data strongly supports the rationale for targeting BET proteins as a new therapeutic strategy that can enhance the efficacy of current therapies.
