# Supplementary material for: BET inhibition as a single or combined therapeutic approach in primary paediatric B-precursor acute lymphoblastic leukaemia
Source: Blood Cancer J. 2013 Jul 19;3(7):e126–. doi: 10.1038/bcj.2013.24 (PMC3730202; doi:10.1038/bcj.2013.24)
Supplement: Supplementary Information [file bcj201324x1.doc]

**Supplementary data:**

**Table 1. Clinical characteristics of primary leukaemia samples analysed**

in this study and JQ1 EC50 values

| Sample | Age at diagnosis  (years) | Clinical stratification  MRD | Cytogenetic profile | WBC  (x10^9^/l) | *In vitro* response to JQ1(μM)  (EC50) |
| --- | --- | --- | --- | --- | --- |
| AML1 | 4.4 | AML | 46XY, t(9;22) | 46.3 | 0.0588 |
| ALL-105 | 2 | HR ALL | 45xy, dic (9;20)+8, -13 | 391.3 | 0.1061 |
| ALL129 | 2.3 | NK | 47,XX,+X,t(3;22)(p25;q13)[9]/  46,XX[1] | 87.8 | 0.1175 |
| ALL-114 | 0.7 | Infant ALL | MLL - rearrangement | 394 | 0.1669 |
| ALL-104 | 6.4 | LR ALL | 46XYt(12;21) | 5.6 | 0.1821 |
| ALL131 | KN | NK | NK | NK | 0.4156 |
| ALL-115 | 11 | LR ALL | 47-48XX | 26.2 | 0.4949 |
| ALL-113 | 1.9 | HR ALL | ND | 10.7 | 0.7242 |
| ALL-108 | 6.4 | LR ALL | 46XY, t(12;21) | 0.6 | 0.725 |
| ALL-111 | 2.7 | LR ALL | 55XY, high hyperdiploid | 42.9 | 0.7921 |
| ALL132 | 9 | NK | t(1;19) | 24.4 | 1.1712 |
| ALL130 | 7.45 | ND | 55,XX,+X,ins(1;?)(q2;?),+4,+6,+10,+14,+17,+18,+21,+21[9]/  46,XX[1] | 40.7 | 1.4495 |
| ALL-122 | 3.6 | HR ALL | 46XY, t(12;21) | 3.4 | 1.4546 |
| ALL-126 | 9 | Relapsed ALL | Hyperdiploid | NK | 1.4719 |
| ALL-101 | 5 | HR ALL | 46 XY | 44.9 | 1.7992 |
| ALL-118 | 1 | Infant ALL | MLL rearrangement | NK | 2.0011 |
| ALL133 | 11 | ND | t(1;19) | 72.6 | 2.6415 |
| ALL-121 | 2 | HR ALL | Hyperdiploid | NK | 2.8095 |
| ALL-110 | 2.5 | HR ALL | 46XY, t(12;21) | 7.5 | 3.3822 |
| AML2 | 7.6 | AML | 46XY, t(15;17) | 6.5 | 3.7202 |
| ALL-112 | 5.5 | HR ALL | Hyperdiploid | 6.5 | 4.3033 |
| ALL-117 | 1.7 | HR ALL | 52XY,+X+4+6+7+8+21(2), 46XY(8) | 56 | 5.7504 |
| ALL-116 | 6.6 | HR ALL | 46XY, t(12;21) | 5.4 | 5.8168 |
| ALL-106 | 2.7 | HR ALL | 46XY del(1)p332p32, dup(2)q2q3),add(9)p2 | 55.9 | 5.8286 |
| ALL-124 | 11 | LR ALL | 46XY, t(12;21) | NK | 19.352 |
| ALL-109 | 13 | LR ALL | 46XY | 2.7 | 20.218 |
| ALL-103 | 5.3 | LR ALL | Hyperdiploid | 3.1 | 23.076 |
| ALL-123 | 2.6 | HR ALL | 54XX +X+4+6+14+17+18+21+21 | 12.1 | 34.172 |

Note: Minimal residual disease was determined by molecular analysis of immune system gene rearrangements. High risk (HR) MRD refers to presence of >5x10^-5^ blast cells and low risk (LR) to ≤ 5x10^-5^ blast cells at day 28 of treatment. WBC= white blood count. EC50 values to 48h incubation with rising concentrations of JQ1 is calculated from the fraction of viable cells affected using Calcusyn Version 2.1for Windows software (Biosoft); NK=not known.

**Figure 1.** **JQ1 extends event free survival and diminishes the majority of ALL subpopulations**

A) Kaplan-Meier plot of event free survival (EFS) based on the time for subcutaneous NALM-6 tumours to attain a size of 1500mm^3^ reveals an extended survival advantage of JQ1 treatment.

B) FACS analysis of hCD45 engraftment levels in peripheral blood of the primary ALL-105 indicates that JQ-1 treatment confers an extended EFS (≥25% hCD45+ engraftment) advantage of at least 24 days. Data are presented as mean ± sd.

Note: *p≤0.01, **p≤0.005, unpaired, two-tailed, students t-test.

C) FACS analysis of the engraftment levels of various ALL subpopulations expressing human CD45 (as a percentage of total live splenic cells) in murine spleens engrafted with the primary sample, ALL-105. This revealed that JQ1-treated animals had reduced levels of the ALL subpopulations, CD34+CD19+CD10-, CD34+CD19+CD10+, CD34-CD19+CD10+ along with a concommitant, relative increase in the CD34-CD19+CD10- population. The remainder of the cells are of murine origin. Data are presented as mean ± sd.

Note: *p≤0.01, **p≤0.005, unpaired, two-tailed, students t-test.

**Table 2. 398 genes ranked according to the EC50 values of 8 primary ALL samples to JQ1**

| Ensembl_gene | Symbol | EC50.spearman.coefficient |
| --- | --- | --- |
| ENSG00000196396 | PTPN1 | 1 |
| ENSG00000240877 | AC073422.1 | 0.976190476 |
| ENSG00000243968 | AD000864.1 | 0.976190476 |
| ENSG00000198944 | ANKRD43 | 0.976190476 |
| ENSG00000172530 | BANP | 0.976190476 |
| ENSG00000156500 | FAM122C | 0.976190476 |
| ENSG00000184903 | IMMP2L | 0.976190476 |
| ENSG00000196976 | LAGE3 | 0.976190476 |
| ENSG00000094661 | OR1I1 | 0.976190476 |
| ENSG00000231925 | TAPBP | 0.976190476 |
| ENSG00000252013 | U4atac | 0.976190476 |
| ENSG00000122042 | UBL3 | 0.976190476 |
| ENSG00000202515 | VTRNA1-3 | 0.976190476 |
| ENSG00000226121 | AC009487.6 | 0.952380952 |
| ENSG00000101544 | ADNP2 | 0.952380952 |
| ENSG00000204673 | AKT1S1 | 0.952380952 |
| ENSG00000124227 | ANKRD60 | 0.952380952 |
| ENSG00000123685 | BATF3 | 0.952380952 |
| ENSG00000187889 | C1orf168 | 0.952380952 |
| ENSG00000255112 | CHMP1B | 0.952380952 |
| ENSG00000198730 | CTR9 | 0.952380952 |
| ENSG00000155744 | FAM126B | 0.952380952 |
| ENSG00000127418 | FGFRL1 | 0.952380952 |
| ENSG00000154252 | GAL3ST2 | 0.952380952 |
| ENSG00000120436 | GPR31 | 0.952380952 |
| ENSG00000170445 | HARS | 0.952380952 |
| ENSG00000092199 | HNRNPC | 0.952380952 |
| ENSG00000169495 | HTRA4 | 0.952380952 |
| ENSG00000116014 | KISS1R | 0.952380952 |
| ENSG00000180660 | MAB21L1 | 0.952380952 |
| ENSG00000183305 | MAGEA2B | 0.952380952 |
| ENSG00000165195 | PIGA | 0.952380952 |
| ENSG00000233746 | RP4-737E23.4 | 0.952380952 |
| ENSG00000085721 | RRN3 | 0.952380952 |
| ENSG00000238729 | snoU13 | 0.952380952 |
| ENSG00000006327 | TNFRSF12A | 0.952380952 |
| ENSG00000140830 | TXNL4B | 0.952380952 |
| ENSG00000201076 | U4 | 0.952380952 |
| ENSG00000172350 | ABCG4 | 0.928571429 |
| ENSG00000240239 | AL158801.1 | 0.928571429 |
| ENSG00000239942 | AL356608.1 | 0.928571429 |
| ENSG00000225637 | AP001046.6 | 0.928571429 |
| ENSG00000143443 | C1orf56 | 0.928571429 |
| ENSG00000159374 | C2orf65 | 0.928571429 |
| ENSG00000171310 | CHST11 | 0.928571429 |
| ENSG00000105443 | CYTH2 | 0.928571429 |
| ENSG00000105821 | DNAJC2 | 0.928571429 |
| ENSG00000179085 | DPM3 | 0.928571429 |
| ENSG00000102890 | ELMO3 | 0.928571429 |
| ENSG00000141971 | FAM125A | 0.928571429 |
| ENSG00000175592 | FOSL1 | 0.928571429 |
| ENSG00000139572 | GPR84 | 0.928571429 |
| ENSG00000147050 | KDM6A | 0.928571429 |
| ENSG00000108510 | MED13 | 0.928571429 |
| ENSG00000171487 | NLRP5 | 0.928571429 |
| ENSG00000153234 | NR4A2 | 0.928571429 |
| ENSG00000011422 | PLAUR | 0.928571429 |
| ENSG00000112033 | PPARD | 0.928571429 |
| ENSG00000124226 | RNF114 | 0.928571429 |
| ENSG00000132874 | SLC14A2 | 0.928571429 |
| ENSG00000200975 | U1 | 0.928571429 |
| ENSG00000212434 | U3 | 0.928571429 |
| ENSG00000117143 | UAP1 | 0.928571429 |
| ENSG00000105668 | UPK1A | 0.928571429 |
| ENSG00000200754 | Y_RNA | 0.928571429 |
| ENSG00000136758 | YME1L1 | 0.928571429 |
| ENSG00000222346 | 5S_rRNA | 0.904761905 |
| ENSG00000222783 | 7SK | 0.904761905 |
| ENSG00000150594 | ADRA2A | 0.904761905 |
| ENSG00000244678 | AP000446.1 | 0.904761905 |
| ENSG00000137727 | ARHGAP20 | 0.904761905 |
| ENSG00000213760 | ATP6V1G2 | 0.904761905 |
| ENSG00000105146 | AURKC | 0.904761905 |
| ENSG00000125378 | BMP4 | 0.904761905 |
| ENSG00000183019 | C19orf59 | 0.904761905 |
| ENSG00000174038 | C9orf131 | 0.904761905 |
| ENSG00000167851 | CD300A | 0.904761905 |
| ENSG00000124762 | CDKN1A | 0.904761905 |
| ENSG00000243649 | CFB | 0.904761905 |
| ENSG00000165215 | CLDN3 | 0.904761905 |
| ENSG00000095794 | CREM | 0.904761905 |
| ENSG00000080007 | DDX43 | 0.904761905 |
| ENSG00000168350 | DEGS2 | 0.904761905 |
| ENSG00000133059 | DSTYK | 0.904761905 |
| ENSG00000138166 | DUSP5 | 0.904761905 |
| ENSG00000117036 | ETV3 | 0.904761905 |
| ENSG00000092820 | EZR | 0.904761905 |
| ENSG00000143340 | FAM163A | 0.904761905 |
| ENSG00000100226 | GTPBP1 | 0.904761905 |
| ENSG00000169045 | HNRNPH1 | 0.904761905 |
| ENSG00000205100 | HSP90AA4P | 0.904761905 |
| ENSG00000137331 | IER3 | 0.904761905 |
| ENSG00000110944 | IL23A | 0.904761905 |
| ENSG00000174010 | KLHL15 | 0.904761905 |
| ENSG00000188581 | KRTAP1-1 | 0.904761905 |
| ENSG00000196569 | LAMA2 | 0.904761905 |
| ENSG00000204428 | LY6G5C | 0.904761905 |
| ENSG00000176624 | MEX3C | 0.904761905 |
| ENSG00000179817 | MRGPRX4 | 0.904761905 |
| ENSG00000188786 | MTF1 | 0.904761905 |
| ENSG00000185619 | PCGF3 | 0.904761905 |
| ENSG00000141934 | PPAP2C | 0.904761905 |
| ENSG00000158615 | PPP1R15B | 0.904761905 |
| ENSG00000160013 | PTGIR | 0.904761905 |
| ENSG00000162924 | REL | 0.904761905 |
| ENSG00000116741 | RGS2 | 0.904761905 |
| ENSG00000143622 | RIT1 | 0.904761905 |
| ENSG00000228856 | RP11-1396O13.11 | 0.904761905 |
| ENSG00000230430 | RP11-1396O13.4 | 0.904761905 |
| ENSG00000229579 | RP11-1396O13.5 | 0.904761905 |
| ENSG00000235780 | RP11-1396O13.7 | 0.904761905 |
| ENSG00000231051 | RP11-1396O13.8 | 0.904761905 |
| ENSG00000231637 | RP11-1396O13.9 | 0.904761905 |
| ENSG00000255384 | RP11-770J1.4 | 0.904761905 |
| ENSG00000118181 | RPS25 | 0.904761905 |
| ENSG00000156304 | SCAF4 | 0.904761905 |
| ENSG00000161055 | SCGB3A1 | 0.904761905 |
| ENSG00000131370 | SH3BP5 | 0.904761905 |
| ENSG00000164638 | SLC29A4 | 0.904761905 |
| ENSG00000199477 | SNORA31 | 0.904761905 |
| ENSG00000196406 | SPANXD | 0.904761905 |
| ENSG00000213246 | SUPT4H1 | 0.904761905 |
| ENSG00000109111 | SUPT6H | 0.904761905 |
| ENSG00000099994 | SUSD2 | 0.904761905 |
| ENSG00000252620 | U6atac | 0.904761905 |
| ENSG00000232264 | USP17 | 0.904761905 |
| ENSG00000227140 | USP17L5 | 0.904761905 |
| ENSG00000126756 | UXT | 0.904761905 |
| ENSG00000175877 | WBSCR28 | 0.904761905 |
| ENSG00000240551 | AC016559.1 | 0.880952381 |
| ENSG00000240909 | AC113407.5 | 0.880952381 |
| ENSG00000240686 | AC124066.1 | 0.880952381 |
| ENSG00000240234 | AC131055.5 | 0.880952381 |
| ENSG00000023330 | ALAS1 | 0.880952381 |
| ENSG00000161618 | ALDH16A1 | 0.880952381 |
| ENSG00000163283 | ALPP | 0.880952381 |
| ENSG00000101246 | ARFRP1 | 0.880952381 |
| ENSG00000125492 | BARHL1 | 0.880952381 |
| ENSG00000165685 | C12orf59 | 0.880952381 |
| ENSG00000089916 | C14orf118 | 0.880952381 |
| ENSG00000162398 | C1orf177 | 0.880952381 |
| ENSG00000186453 | C2orf84 | 0.880952381 |
| ENSG00000196821 | C6orf106 | 0.880952381 |
| ENSG00000197191 | C9orf169 | 0.880952381 |
| ENSG00000136805 | C9orf4 | 0.880952381 |
| ENSG00000178243 | C9orf62 | 0.880952381 |
| ENSG00000129048 | CCRL1 | 0.880952381 |
| ENSG00000111642 | CHD4 | 0.880952381 |
| ENSG00000100888 | CHD8 | 0.880952381 |
| ENSG00000184113 | CLDN5 | 0.880952381 |
| ENSG00000163254 | CRYGC | 0.880952381 |
| ENSG00000226372 | DCAF8L1 | 0.880952381 |
| ENSG00000142025 | DMRTC2 | 0.880952381 |
| ENSG00000069345 | DNAJA2 | 0.880952381 |
| ENSG00000137094 | DNAJB5 | 0.880952381 |
| ENSG00000104885 | DOT1L | 0.880952381 |
| ENSG00000000419 | DPM1 | 0.880952381 |
| ENSG00000157540 | DYRK1A | 0.880952381 |
| ENSG00000141543 | EIF4A3 | 0.880952381 |
| ENSG00000075426 | FOSL2 | 0.880952381 |
| ENSG00000205097 | FRG2 | 0.880952381 |
| ENSG00000225899 | FRG2B | 0.880952381 |
| ENSG00000224659 | GAGE12J | 0.880952381 |
| ENSG00000166123 | GPT2 | 0.880952381 |
| ENSG00000137947 | GTF2B | 0.880952381 |
| ENSG00000113924 | HGD | 0.880952381 |
| ENSG00000164508 | HIST1H2AA | 0.880952381 |
| ENSG00000235734 | HMGN1P36 | 0.880952381 |
| ENSG00000095752 | IL11 | 0.880952381 |
| ENSG00000167378 | IRGQ | 0.880952381 |
| ENSG00000240871 | KRTAP4-7 | 0.880952381 |
| ENSG00000167470 | MIDN | 0.880952381 |
| ENSG00000207933 | MIR9-1 | 0.880952381 |
| ENSG00000243696 | MUSTN1 | 0.880952381 |
| ENSG00000167604 | NFKBID | 0.880952381 |
| ENSG00000116962 | NID1 | 0.880952381 |
| ENSG00000146909 | NOM1 | 0.880952381 |
| ENSG00000175352 | NRIP3 | 0.880952381 |
| ENSG00000170683 | OR10A3 | 0.880952381 |
| ENSG00000176904 | OR51H1P | 0.880952381 |
| ENSG00000174957 | OR5J2 | 0.880952381 |
| ENSG00000179094 | PER1 | 0.880952381 |
| ENSG00000186866 | POFUT2 | 0.880952381 |
| ENSG00000100227 | POLDIP3 | 0.880952381 |
| ENSG00000255963 | PPIAL4A | 0.880952381 |
| ENSG00000142538 | PTH2 | 0.880952381 |
| ENSG00000112245 | PTP4A1 | 0.880952381 |
| ENSG00000129472 | RAB2B | 0.880952381 |
| ENSG00000102317 | RBM3 | 0.880952381 |
| ENSG00000160439 | RDH13 | 0.880952381 |
| ENSG00000165799 | RNASE7 | 0.880952381 |
| ENSG00000214062 | RP11-1E1.1 | 0.880952381 |
| ENSG00000223738 | RP11-781E19.1 | 0.880952381 |
| ENSG00000161526 | SAP30BP | 0.880952381 |
| ENSG00000176994 | SMCR8 | 0.880952381 |
| ENSG00000206602 | SNORD58A | 0.880952381 |
| ENSG00000149922 | TBX6 | 0.880952381 |
| ENSG00000163060 | TEKT4 | 0.880952381 |
| ENSG00000177370 | TIMM22 | 0.880952381 |
| ENSG00000157150 | TIMP4 | 0.880952381 |
| ENSG00000142484 | TM4SF5 | 0.880952381 |
| ENSG00000183864 | TOB2 | 0.880952381 |
| ENSG00000101150 | TPD52L2 | 0.880952381 |
| ENSG00000222612 | U2 | 0.880952381 |
| ENSG00000092929 | UNC13D | 0.880952381 |
| ENSG00000170185 | USP38 | 0.880952381 |
| ENSG00000197279 | ZNF165 | 0.880952381 |
| ENSG00000249034 | AC005609.17 | -0.880952381 |
| ENSG00000148700 | ADD3 | -0.880952381 |
| ENSG00000159063 | ALG8 | -0.880952381 |
| ENSG00000185722 | ANKFY1 | -0.880952381 |
| ENSG00000198826 | ARHGAP11A | -0.880952381 |
| ENSG00000197299 | BLM | -0.880952381 |
| ENSG00000184992 | BRI3BP | -0.880952381 |
| ENSG00000162384 | C1orf123 | -0.880952381 |
| ENSG00000183323 | CCDC125 | -0.880952381 |
| ENSG00000119929 | CUTC | -0.880952381 |
| ENSG00000204548 | DEFB121 | -0.880952381 |
| ENSG00000035499 | DEPDC1B | -0.880952381 |
| ENSG00000205981 | DNAJC19 | -0.880952381 |
| ENSG00000172269 | DPAGT1 | -0.880952381 |
| ENSG00000128829 | EIF2AK4 | -0.880952381 |
| ENSG00000158483 | FAM86C1 | -0.880952381 |
| ENSG00000144554 | FANCD2 | -0.880952381 |
| ENSG00000140525 | FANCI | -0.880952381 |
| ENSG00000125447 | GGA3 | -0.880952381 |
| ENSG00000115207 | GTF3C2 | -0.880952381 |
| ENSG00000138668 | HNRNPD | -0.880952381 |
| ENSG00000133835 | HSD17B4 | -0.880952381 |
| ENSG00000067704 | IARS2 | -0.880952381 |
| ENSG00000164941 | INTS8 | -0.880952381 |
| ENSG00000171121 | KCNMB3 | -0.880952381 |
| ENSG00000120733 | KDM3B | -0.880952381 |
| ENSG00000121621 | KIF18A | -0.880952381 |
| ENSG00000105486 | LIG1 | -0.880952381 |
| ENSG00000107798 | LIPA | -0.880952381 |
| ENSG00000113368 | LMNB1 | -0.880952381 |
| ENSG00000118308 | LRMP | -0.880952381 |
| ENSG00000102897 | LYRM1 | -0.880952381 |
| ENSG00000172469 | MANEA | -0.880952381 |
| ENSG00000104814 | MAP4K1 | -0.880952381 |
| ENSG00000078070 | MCCC1 | -0.880952381 |
| ENSG00000124370 | MCEE | -0.880952381 |
| ENSG00000111554 | MDM1 | -0.880952381 |
| ENSG00000122033 | MTIF3 | -0.880952381 |
| ENSG00000063601 | MTMR1 | -0.880952381 |
| ENSG00000178057 | NDUFAF3 | -0.880952381 |
| ENSG00000151092 | NGLY1 | -0.880952381 |
| ENSG00000152465 | NMT2 | -0.880952381 |
| ENSG00000167005 | NUDT21 | -0.880952381 |
| ENSG00000132356 | PRKAA1 | -0.880952381 |
| ENSG00000213005 | PTTG3P | -0.880952381 |
| ENSG00000103490 | PYCARD | -0.880952381 |
| ENSG00000029725 | RABEP1 | -0.880952381 |
| ENSG00000080298 | RFX3 | -0.880952381 |
| ENSG00000120925 | RNF170 | -0.880952381 |
| ENSG00000143653 | SCCPDH | -0.880952381 |
| ENSG00000103064 | SLC7A6 | -0.880952381 |
| ENSG00000163029 | SMC6 | -0.880952381 |
| ENSG00000165209 | STRBP | -0.880952381 |
| ENSG00000108264 | TADA2A | -0.880952381 |
| ENSG00000172660 | TAF15 | -0.880952381 |
| ENSG00000154743 | TSEN2 | -0.880952381 |
| ENSG00000018699 | TTC27 | -0.880952381 |
| ENSG00000184076 | UQCR10 | -0.880952381 |
| ENSG00000136936 | XPA | -0.880952381 |
| ENSG00000134308 | YWHAQ | -0.880952381 |
| ENSG00000142065 | ZFP14 | -0.880952381 |
| ENSG00000215068 | AC025171.1 | -0.904761905 |
| ENSG00000243651 | AC115220.8 | -0.904761905 |
| ENSG00000154930 | ACSS1 | -0.904761905 |
| ENSG00000168803 | ADAL | -0.904761905 |
| ENSG00000197049 | AL669831.1 | -0.904761905 |
| ENSG00000241468 | ATP5J2 | -0.904761905 |
| ENSG00000196559 | C11orf55 | -0.904761905 |
| ENSG00000184220 | C3orf26 | -0.904761905 |
| ENSG00000197603 | C5orf42 | -0.904761905 |
| ENSG00000151065 | DCP1B | -0.904761905 |
| ENSG00000162894 | FAIM3 | -0.904761905 |
| ENSG00000166801 | FAM111A | -0.904761905 |
| ENSG00000077458 | FAM76B | -0.904761905 |
| ENSG00000174500 | GCET2 | -0.904761905 |
| ENSG00000076650 | GPATCH1 | -0.904761905 |
| ENSG00000122034 | GTF3A | -0.904761905 |
| ENSG00000128731 | HERC2 | -0.904761905 |
| ENSG00000180596 | HIST1H2BC | -0.904761905 |
| ENSG00000071794 | HLTF | -0.904761905 |
| ENSG00000211958 | IGHV3-38 | -0.904761905 |
| ENSG00000138795 | LEF1 | -0.904761905 |
| ENSG00000104863 | LIN7B | -0.904761905 |
| ENSG00000154589 | LY96 | -0.904761905 |
| ENSG00000197442 | MAP3K5 | -0.904761905 |
| ENSG00000051825 | MPHOSPH9 | -0.904761905 |
| ENSG00000166902 | MRPL16 | -0.904761905 |
| ENSG00000123338 | NCKAP1L | -0.904761905 |
| ENSG00000140396 | NCOA2 | -0.904761905 |
| ENSG00000174886 | NDUFA11 | -0.904761905 |
| ENSG00000184752 | NDUFA12 | -0.904761905 |
| ENSG00000140694 | PARN | -0.904761905 |
| ENSG00000143799 | PARP1 | -0.904761905 |
| ENSG00000126249 | PDCD2L | -0.904761905 |
| ENSG00000145287 | PLAC8 | -0.904761905 |
| ENSG00000100362 | PVALB | -0.904761905 |
| ENSG00000161800 | RACGAP1 | -0.904761905 |
| ENSG00000026297 | RNASET2 | -0.904761905 |
| ENSG00000108375 | RNF43 | -0.904761905 |
| ENSG00000258608 | RP11-35B20.1 | -0.904761905 |
| ENSG00000185495 | RP11-504P24.4 | -0.904761905 |
| ENSG00000197185 | RP11-552J9.5 | -0.904761905 |
| ENSG00000183291 | RP4-604K5.1 | -0.904761905 |
| ENSG00000132383 | RPA1 | -0.904761905 |
| ENSG00000178217 | SH2D4B | -0.904761905 |
| ENSG00000108528 | SLC25A11 | -0.904761905 |
| ENSG00000075303 | SLC25A40 | -0.904761905 |
| ENSG00000172716 | SLFN11 | -0.904761905 |
| ENSG00000139613 | SMARCC2 | -0.904761905 |
| ENSG00000099940 | SNAP29 | -0.904761905 |
| ENSG00000186767 | SPIN4 | -0.904761905 |
| ENSG00000127952 | STYXL1 | -0.904761905 |
| ENSG00000111843 | TMEM14C | -0.904761905 |
| ENSG00000140455 | USP3 | -0.904761905 |
| ENSG00000129204 | USP6 | -0.904761905 |
| ENSG00000111237 | VPS29 | -0.904761905 |
| ENSG00000188177 | ZC3H6 | -0.904761905 |
| ENSG00000135847 | ACBD6 | -0.928571429 |
| ENSG00000242110 | AMACR | -0.928571429 |
| ENSG00000140750 | ARHGAP17 | -0.928571429 |
| ENSG00000119397 | CNTRL | -0.928571429 |
| ENSG00000172795 | DCP2 | -0.928571429 |
| ENSG00000228716 | DHFR | -0.928571429 |
| ENSG00000128951 | DUT | -0.928571429 |
| ENSG00000147475 | ERLIN2 | -0.928571429 |
| ENSG00000185104 | FAF1 | -0.928571429 |
| ENSG00000112234 | FBXL4 | -0.928571429 |
| ENSG00000168522 | FNTA | -0.928571429 |
| ENSG00000077809 | GTF2I | -0.928571429 |
| ENSG00000206149 | HERC2P9 | -0.928571429 |
| ENSG00000072571 | HMMR | -0.928571429 |
| ENSG00000171401 | KRT13 | -0.928571429 |
| ENSG00000066855 | MTFR1 | -0.928571429 |
| ENSG00000102699 | PARP4 | -0.928571429 |
| ENSG00000180822 | PSMG4 | -0.928571429 |
| ENSG00000151552 | QDPR | -0.928571429 |
| ENSG00000155906 | RMND1 | -0.928571429 |
| ENSG00000143379 | SETDB1 | -0.928571429 |
| ENSG00000164975 | SNAPC3 | -0.928571429 |
| ENSG00000147687 | TATDN1 | -0.928571429 |
| ENSG00000111602 | TIMELESS | -0.928571429 |
| ENSG00000198586 | TLK1 | -0.928571429 |
| ENSG00000135148 | TRAFD1 | -0.928571429 |
| ENSG00000198677 | TTC37 | -0.928571429 |
| ENSG00000137822 | TUBGCP4 | -0.928571429 |
| ENSG00000129003 | VPS13C | -0.928571429 |
| ENSG00000028116 | VRK2 | -0.928571429 |
| ENSG00000105053 | VRK3 | -0.928571429 |
| ENSG00000058799 | YIPF1 | -0.928571429 |
| ENSG00000147905 | ZCCHC7 | -0.928571429 |
| ENSG00000243015 | AC025260.1 | -0.952380952 |
| ENSG00000117448 | AKR1A1 | -0.952380952 |
| ENSG00000197775 | AL136419.1 | -0.952380952 |
| ENSG00000012779 | ALOX5 | -0.952380952 |
| ENSG00000248546 | ANP32C | -0.952380952 |
| ENSG00000149311 | ATM | -0.952380952 |
| ENSG00000176209 | C8orf40 | -0.952380952 |
| ENSG00000156136 | DCK | -0.952380952 |
| ENSG00000109911 | ELP4 | -0.952380952 |
| ENSG00000164308 | ERAP2 | -0.952380952 |
| ENSG00000154153 | FAM134B | -0.952380952 |
| ENSG00000111913 | FAM65B | -0.952380952 |
| ENSG00000140718 | FTO | -0.952380952 |
| ENSG00000173221 | GLRX | -0.952380952 |
| ENSG00000113648 | H2AFY | -0.952380952 |
| ENSG00000132196 | HSD17B7 | -0.952380952 |
| ENSG00000182054 | IDH2 | -0.952380952 |
| ENSG00000115204 | MPV17 | -0.952380952 |
| ENSG00000120526 | NUDCD1 | -0.952380952 |
| ENSG00000064933 | PMS1 | -0.952380952 |
| ENSG00000169413 | RNASE6 | -0.952380952 |
| ENSG00000136169 | SETDB2 | -0.952380952 |
| ENSG00000103266 | STUB1 | -0.952380952 |
| ENSG00000170310 | STX8 | -0.952380952 |
| ENSG00000163634 | THOC7 | -0.952380952 |
| ENSG00000172765 | TMCC1 | -0.952380952 |
| ENSG00000118640 | VAMP8 | -0.952380952 |
| ENSG00000255604 | VTN | -0.952380952 |
| ENSG00000165392 | WRN | -0.952380952 |
| ENSG00000254837 | AP001372.2 | -0.976190476 |
| ENSG00000188186 | C7orf59 | -0.976190476 |
| ENSG00000151849 | CENPJ | -0.976190476 |
| ENSG00000166797 | FAM96A | -0.976190476 |
| ENSG00000147592 | LACTB2 | -0.976190476 |
| ENSG00000110536 | PTPMT1 | -0.976190476 |
| ENSG00000077097 | TOP2B | -0.976190476 |
| ENSG00000067167 | TRAM1 | -0.976190476 |
| ENSG00000163872 | YEATS2 | -0.976190476 |
| ENSG00000179456 | ZNF238 | -0.976190476 |
| ENSG00000112486 | CCR6 | -1 |
| ENSG00000198648 | STK39 | -1 |

**Figure 2.** Cytoscape analysis of 398 genes ranked according to EC50 highlighted two major pathways predictive of cytotoxic response of primary ALLs to JQ1: one associated with ATM (white circle) and related DSB repair genes, and the other linked with the cycle inhibitor CDKN1A (black circle).

**Table 3. JQ1-induced transcriptional responses in primary ALL samples** by Gene Set Enrichment Analysis (GSEA)

| **Pathway name** | **Number of genes in the pathway** | **NOM  *P*** | **FDR *q*** |
| --- | --- | --- | --- |
| IL17 PATHWAY | 13 | 0 | 0.01 |
| CELL SURFACE RECEPTOR LINKED SIGNAL TRANSDUCTION | 121 | 0 | 0.01 |
| NFkB PATHWAY | 106 | 0 | 0.08 |
| INFLAMMATORY RESPONSE PATHWAY | 29 | 0.002 | 0.02 |
| CYTOKINE CONNECTION | 15 | 0.02 | 0.1 |
| IL7 PATHWAY | 16 | 0.014 | 0.1 |
| MYC PATHWAY | 7 | 0.038 | 0.1 |
| IL10 PATHWAY | 13 | 0.039 | 0.14 |
| IL2RB PATHWAY | 34 | 0.012 | 0.14 |
| STRESS SPECIFIC | 44 | 0.012 | 0.15 |
| CELL ADHESION | 132 | 0.006 | 0.15 |
| IL1 PATHWAY | 62 | 0.014 | 0.17 |
| JAK/STAT SIGNALING PATHWAY | 150 | 0.01 | 0.2 |
| VEGF | 23 | 0.05 | 0.22 |
| TOLL PATHWAY | 32 | 0.06 | 0.26 |
| BRCA/BRCA1 PATHWAY | 93 | 0.02 | 0.264 |
| CELL PROLIFERATION | 196 | 0.01 | 0.27 |

NOM *P* indicates nominal probability.

**Figure 3. JQ1-induced cell death is mediated by apoptosis**

A) Cellular apoptotic and death levels of the NALM-6 cell line were quantified by Annexin V (AxV)/PI staining according to the manufacturer’s instructions (BD Pharmingen). JQ1 induced significant (p=0.01) apoptotic cell death as evidenced by single AxV staining with minimal PI uptake.

Note: *p≤0.01, paired, two-tailed, students t-test.

B) Furthermore, more detailed western blot analysis of additional ALL cell lines confirmed JQ1 induced apoptosis by demonstrating the synchronous appearance of cleaved PARP1, Caspase-7 and Caspase-3 within 72-96h of exposure to JQ1.

**
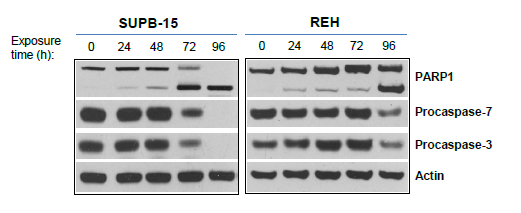
**

**Cleaved PARP1**

**Figure 4. A Venn diagram reveals a lack of common target genes between JQ1 and glucocorticosteroids in paediatric ALL**

The Venny program (<http://bioinfogp.cnb.csic.es/tools/venny/index.html>) was used to generate Venn diagrams to determine common gene targets between JQ1 and glucocorticosteroids (GCs). By comparing JQ1-induced expression profiles in primary ALLs in this study with the available data for the GCs target genes^31^ we identified only a single common gene upregulated by both JQ1 and GCs (FKBP51).

**Tables 4 and 5. Evaluation of synergy between JQ1 and dexamethasone**

Where possible, when cells were sensitive to both reagents, we determined the combination indices of 0.1μM JQ1 co-treatment with rising concentrations of dexamethasone (0.0001-10μM) both in ALL cell lines (NALM-6, TOM-1) (Supplementary table 4) and primary samples (ALL-129, ALL-130, ALL-132) (Supplementary table 5).

To determine a combination index (CI) and evaluate whether individual treatment combinations were synergistic (CI<1), antagonistic (CI>1), or additive (CI=1) we used the Calcusyn program.

Note: +++++ very strong synergism; ++++ strong synergism; +++ synergism; ++ moderate synergism; --- antagonism; ---- strong antagonism; ---- very strong antagonism.

**Table 4**

**Table 5**

**Figure 5. Co-treatment of JQ1 with Dexamethasone prolongs event free survival**

A) Kaplan-Meier plot of event free survival (EFS) based on the time for subcutaneous REH tumours to attain a size of 2500mm^3^ reveals an extended survival advantage of combined JQ1 + dexamethasone treatment.

B) FACS analysis of hCD45 engraftment levels in peripheral blood of the primary ALL-132 indicates that whilst JQ-1 co-treatment with dexamethasone significantly reduces engraftment levels there is no effect upon EFS (≥25% hCD45+ engraftment). We surmise this is because ALL-132 is highly proliferative. However, a survival advantage of this co-treatment can be inferred from the fact that co-treated animals were still healthy at the experimental endpoint as opposed to either the vehicle or single agent-treated animals. Data are presented as mean ± sd.

Note: *p≤0.01, **p≤0.005, unpaired, two-tailed, students t-test.
